# Decoding Prodromal Lewy Body Disease: Clinical Differences Between Isolated REM Sleep Behavior Disorder and Hyposmia With Dopamine Transporter Deficit

**DOI:** 10.1111/ene.70724

**Published:** 2026-08-06

**Authors:** Luke Vikram Banerjee, Jacopo Pasquini, Robin Henderson, Nicola Pavese, Kirstie N. Anderson

**Affiliations:** ^1^ Clinical Ageing Research Unit Newcastle University, Campus for Ageing and Vitality Newcastle upon Tyne UK; ^2^ Department of Clinical and Experimental Medicine University of Pisa Pisa Italy; ^3^ School of Mathematics, Statistics and Physics Newcastle University Newcastle upon Tyne UK; ^4^ Department of Nuclear Medicine and PET Centre Aarhus University Hospital Aarhus Denmark; ^5^ Regional Sleep Service Newcastle Upon Tyne NHS Foundation Trust Newcastle upon Tyne UK

**Keywords:** dementia with Lewy bodies, hyposmia, Parkinson's disease, prodromal, REM sleep behavior disorder

## Abstract

**Background:**

Lewy body diseases (LBDs) are heterogeneous, and this variability is already evident in the prodromal phase. Several frameworks propose that prodromal heterogeneity reflects distinct phenotypic and biological subtypes, supported by differences in clinical profiles, imaging, and α‐synuclein biomarkers. We provide a detailed clinical characterization of two enriched prodromal cohorts: isolated REM Sleep Behavior Disorder (iRBD) and Hyposmia with Dopamine Transporter Deficit.

**Methods:**

This cross‐sectional study included 360 participants with iRBD, 1101 with hyposmia and abnormal dopamine transporter imaging and 283 healthy controls from the Parkinson's Progression Markers Initiative. Using the earliest assessments available, we compared the Montreal Cognitive Assessment (MoCA), Scales for Outcomes in Parkinson's Disease Autonomic Dysfunction (SCOPA‐AUT) and Movement Disorder Society Unified Parkinson's Disease Rating Scale (MDS‐UPDRS) Parts I‐III. Group differences were assessed using age and sex‐adjusted permutation testing. Secondary descriptive analysis identified the individual items contributing most to between‐group differences.

**Results:**

Both prodromal cohorts had significantly worse scores than healthy controls in all assessments. Compared with hyposmia, iRBD was associated with higher SCOPA‐AUT and MDS‐UPDRS Part I scores, driven mainly by constipation and urinary symptoms.

**Conclusion:**

In prodromal Lewy body disease, iRBD is associated with a statistically significant excess of autonomic symptom burden compared with hyposmia with dopamine transporter deficit, with the largest item‐level differences in urinary and constipation measures.

## Graphical abstract

Isolated REM sleep behavior disorder (iRBD) [*n* = 360] and hyposmia with dopamine transporter deficit [*n* = 1101] represent two enriched prodromal Lewy body disease cohorts. Using data from the Parkinson's Progression Markers Initiative, iRBD was associated with significantly greater autonomic symptom burden than hyposmia, driven by constipation and urinary symptoms, while cognitive and motor profiles were similar. These findings support distinct prodromal phenotypes consistent with the brain‐first/body‐first framework.

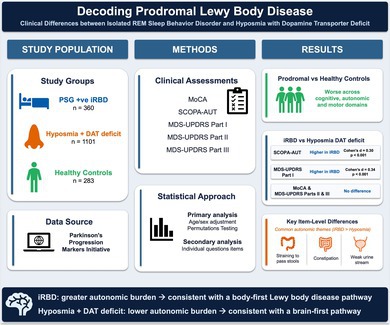

## 1 Introduction

Lewy body disease (LBD) encompasses a disease spectrum that includes Parkinson's disease (PD) and dementia with Lewy bodies (DLB). PD is the fastest growing neurological disorder globally and DLB accounts for approximately 3%–7% of diagnosed dementias, highlighting both as major public health priorities [1, 2]. Both conditions share an overlapping prodromal phase, where neurodegeneration is occurring long before a diagnostic clinical syndrome [3, 4, 5]. In this phase, it is generally not possible to distinguish PD from DLB or other LBDs. The prodrome is highly heterogeneous, with variability in the rate of disease progression, age of onset, and the pattern and severity of symptoms [3, 6, 7]. Understanding this phase remains challenging, in part due to the difficulty of identifying and recruiting patients to research trials before manifestation of overt clinical symptoms.

Studies exploring heterogeneity in prodromal LBD have either been hypothesis‐led, testing predefined subgroups, or data‐driven approaches, using unsupervised clustering [7, 8]. Across numerous recent imaging, clinical marker, and postmortem studies using both methodologies, isolated rapid eye movement sleep behavior disorder (iRBD) emerges as a recurring phenotype across the PD‐DLB spectrum, with iRBD‐positive and iRBD‐negative prodromal presentations commonly described [3, 6, 9, 10, 11]. Recent PPMI analyses suggest that baseline non‐motor symptom profiles in prodromal cohorts can also have prognostic value, predicting both time to PD diagnosis and motor phenotype at diagnosis [12]. However, detailed characterization of between‐group clinical differences at the prodromal stage itself remains limited.

This study aims to characterize early clinical distinctions between two enriched prodromal cohorts: an iRBD‐positive group with polysomnography‐confirmed iRBD and an iRBD‐negative group defined by hyposmia with abnormal Dopamine Transporter Single Photon Emission Computed Tomography (DAT‐SPECT). Both iRBD and hyposmia with DAT deficit are established prodromal markers associated with a high risk of conversion to LBD. As demonstrated in the Parkinson Associated Risk Syndrome study, 67% of hyposmic individuals with DAT deficit converted to PD within 4 years, with high conversion rates confirmed at long‐term follow‐up and in the largest multicentre iRBD study to date, the phenoconversion rate to an overt neurodegenerative syndrome was 6.3% per year, reaching 73.5% at 12 years [13, 14, 15].

We provide a detailed comparison of cognitive, autonomic, and motor profiles between these cohorts in a large prodromal sample (*n* = 1461). Overall, we aim to characterize phenotypic differences between these prodromal cohorts, with implications for understanding prodromal heterogeneity and informing the design of future neuroprotective trials.

## 2 Methods

### 2.1 Data

Data was acquired from the Parkinson's Progression Markers Initiative (PPMI) database downloaded on January 6th, 2025. PPMI is a worldwide, multicenter, longitudinal cohort study, which aims to identify biological markers of PD onset and progression. Data is available at www.ppmi‐info.org/access‐dataspecimens/download‐data, (Research Resource Identifier: RRID SCR_006431). For up‐to‐date information on the study, including cohort recruitment and eligibility criteria, visit www.ppmi‐info.org [16]. This study used tier 1 data, openly available from PPMI, and tier 3 data, obtained from PPMI upon request after approval by the PPMI Data Access Committee.

For each participant assessment, the earliest datapoint available in the dataset was selected. For participants with missing datapoints, we employed a listwise deletion approach.

### 2.2 Population

For all groups, participants with any of the following were excluded from our dataset: diagnosis of PD or DLB, under the age of 50 years, and carriers of known PD genetic variants (Leucine‐Rich Repeat Kinase 2, β‐Glucocerebrosidase, Alpha‐Synuclein, Parkin RBR E3 Ubiquitin Protein Ligase, and PTEN Induced Putative Kinase 1).

Participants with hyposmia were recruited to PPMI if they met two criteria: a score at or below the 15th percentile for age and sex on the University of Pennsylvania Smell Identification Test (UPSIT) and abnormal DAT‐SPECT imaging [17, 18]. An abnormal DAT‐SPECT was defined by a hybrid of expert visual interpretation indicating dopaminergic deficit and quantitative analysis, encompassing both dopaminergic deficit and reduction relative to age‐expected values. For our study, we excluded any hyposmia participants in the PPMI database that had any diagnosis of iRBD (either clinical history or polysomnography confirmed).

Data from participants with polysomnography (PSG) confirmed iRBD was extracted from the PPMI database. iRBD participants were recruited to PPMI from collaborating sleep centers with an existing PSG‐confirmed diagnosis of RBD. We excluded any iRBD participants in the PPMI database that also had a diagnosis of hyposmia. Therefore, there was no overlap between iRBD and hyposmic groups. iRBD participants also underwent DAT‐SPECT scans; the state of the nigrostriatal system was not a requirement for inclusion in this cohort.

Healthy controls were included based on the following criteria: they had no first‐degree relative with a PD diagnosis, no significant neurological disorders, and a visually normal DAT‐SPECT.

### 2.3 Clinical Assessments

We obtained the following assessments for each participant: the Montreal Cognitive Assessment (MoCA), the Scales for Outcomes in Parkinson's Disease Autonomic Dysfunction (SCOPA‐AUT), and the Movement Disorder Society Unified Parkinson's Disease Rating Scale (MDS‐UPDRS) parts I‐III [19, 20, 21]. SCOPA‐AUT sex‐related questions were omitted from our analysis, as a significant proportion of participants across all subgroups selected ‘not applicable’ in response to these questions.

### 2.4 Statistical Analysis

Data were pre‐processed in Python v3 and analyzed in R studio. The primary analysis compared mean clinical scores between groups. To adjust for the potentially confounding variables age and sex, we used a two‐stage residual adjustment approach [22]. First, we constructed a linear model regressing each score on age and sex using all participants (iRBD, hyposmia, controls), with no group term in this model. Second, we tested for between‐group differences using the resulting residuals, thereby assessing group effects after adjustment for age and sex.

Permutation testing (10,000 permutations) was employed to evaluate the statistical significance of differences observed between groups by shuffling group labels on the age‐ and sex‐adjusted residuals [23]. This non‐parametric approach was chosen because responses were discrete and skewed. To compare the three groups whilst maintaining independence between tests, we performed two non‐overlapping comparisons: (1) the combined prodromal group (iRBD and hyposmia) versus healthy controls and (2) a comparison between prodromal groups. All tests were two‐sided.

Statistical significance was set a priori at *p* < 0.05. To address multiplicity from testing five outcomes within each primary comparison (MoCA, SCOPA‐AUT, MDS‐UPDRS I‐III), we applied a Bonferroni correction (*p* < 0.01 per outcome). Effect sizes were reported as Cohen's d, calculated as the difference in age‐ and sex‐adjusted mean scores between groups divided by the residual standard deviation (the square root of the model's mean squared residual error) from the age/sex adjustment model fitted in the full sample (iRBD, hyposmia, and controls).

We performed a descriptive secondary analysis by plotting mean differences and confidence intervals for individual question responses. This approach allowed us to identify which questions were driving the overall group differences in assessment scores. Individual question differences were not subject to hypothesis testing, as the identification of significant differences in single items was not relevant for the overall scope of the analysis and would have introduced a high risk of Type I error due to multiple comparisons.

## 3 Results

**FIGURE 1:**
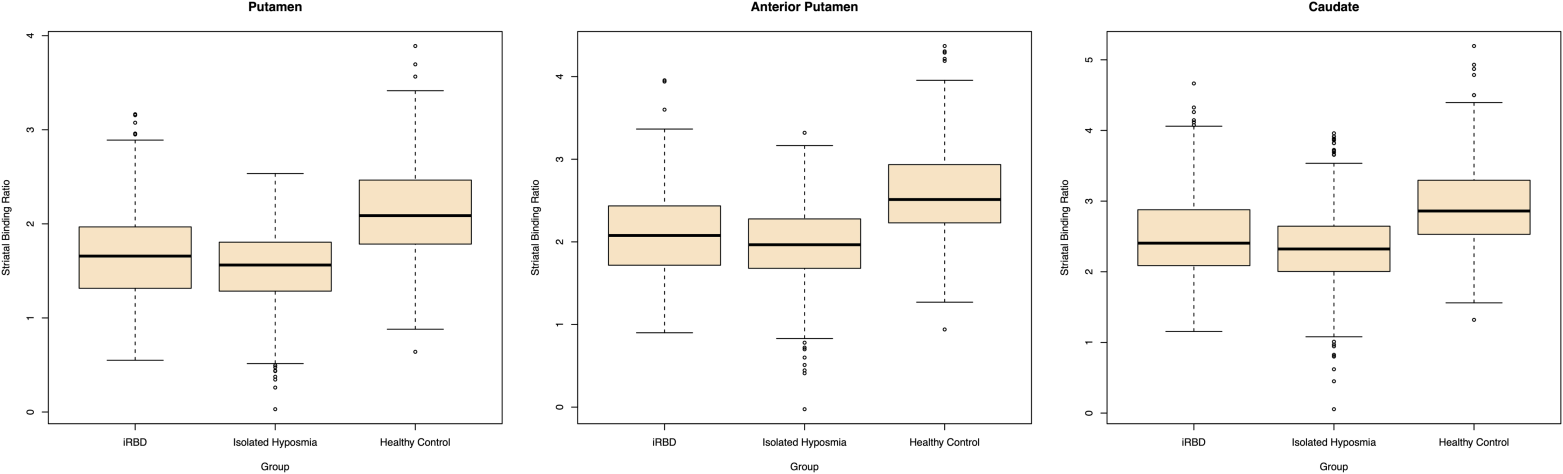
Boxplots to show DAT‐SPECT striatal binding ratios by group and brain region.

List‐wise deletion resulted in the exclusion of 92 participants with incomplete data: 19 iRBD, 70 hyposmia, and 3 healthy controls, leaving 1744 participants for the primary analyses (Table 1). The two prodromal cohorts were of similar age and both were older than controls. The iRBD group was predominantly male, consistent with current evidence on sex distribution in iRBD [24], and the median duration of iRBD symptoms prior to enrolment was 6.1 years (mean 9.1, SD 9.8; data available for 347/360 participants). Figure 1 summarizes regional DAT‐SPECT binding by group; as expected, values reflected enrichment for dopaminergic deficit in the hyposmia/DAT‐deficit cohort relative to controls.

**TABLE 1:** Participant characteristics.

Characteristic	iRBD (*n* = 360)	Hyposmic (*n* = 1101)	Healthy Controls (*n* = 283)	*p* ^b^
Age at Visit (years)	67.7 (6.1)	67.9 (5.5)	65.3 (7.9)	< 0.001
Sex	78 females	670 females	114 females	< 0.001
	282 males	431 males	169 males	
Years in Education	16.3 (3.5)	16.7 (2.8)	16.5 (3.0)	0.027
MoCA	26.3 (2.8)	26.7 (2.3)	27.8 (1.6)	< 0.001
SCOPA	10.6 (6.8)	9.3 (6.1)	5.7 (3.9)	< 0.001
MDS‐UPDRS Part 1	8.0 (5.7)	6.8 (5.0)	3.2 (3.0)	< 0.001
MDS‐UPDRS Part 2	2.4 (3.4)	2.2 (3.5)	0.5 (1.0)	< 0.001
MDS‐UPDRS Part 3	4.6 (5.3)	5.0 (6.3)	1.6 (2.4)	< 0.001
Putamen SBR^a^	1.66 (0.52)	1.52 (0.39)	2.13 (0.50)	< 0.001
Anterior Putamen SBR^a^	2.09 (0.56)	1.95 (0.45)	2.60 (0.54)	< 0.001
Caudate SBR^a^	2.48 (0.60)	2.33 (0.51)	2.95 (0.60)	< 0.001

*Note:* Data values appear as unadjusted means (standard deviation).

^a^ Striatal binding ratio (SBR) is a semi‐quantitative measure calculated from DAT‐SPECT data. It compares tracer uptake in the area of interest to that in a low‐uptake reference tissue (occipital cortex). SBR was calculated as a mean across both hemispheres. Data values for DAT‐SPECT were missing for 16 iRBD participants, one hyposmia participant and 13 healthy control participants. Missing participants were excluded from DAT‐SPECT mean and standard deviation calculations but otherwise had complete datasets.

^b^ *p‐*values from one‐way ANOVA (continuous variables) or Pearson's chi‐squared test (sex).

**FIGURE 2:**
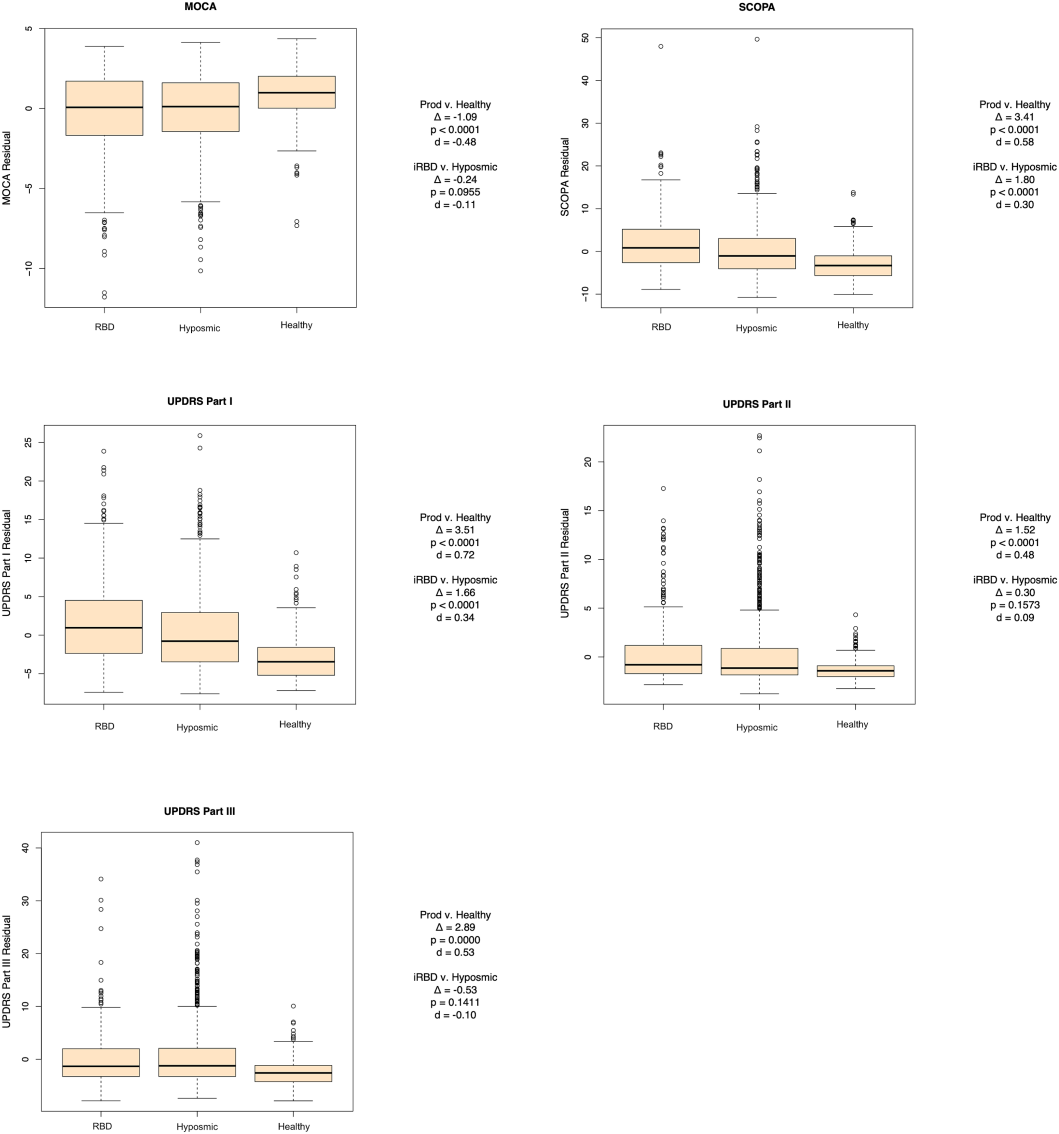
Boxplots of age‐ and sex‐adjusted residuals for each outcome by group. *p* values are Bonferroni‐corrected using cut off *p* < 0.01 within each primary comparison. Δ indicates the adjusted mean difference (first group minus comparator); d indicates Cohen's d.

The combined prodromal cohort performed worse than healthy controls across all outcomes, with small‐to‐moderate effect sizes. Within the prodromal cohorts, iRBD was associated with a modest excess of autonomic and non‐motor symptom burden (SCOPA‐AUT and MDS‐UPDRS Part I), while cognition (MoCA) and motor severity (MDS‐UPDRS Parts II–III) did not differ materially between iRBD and hyposmia groups (Figure 2).

**FIGURE 3:**
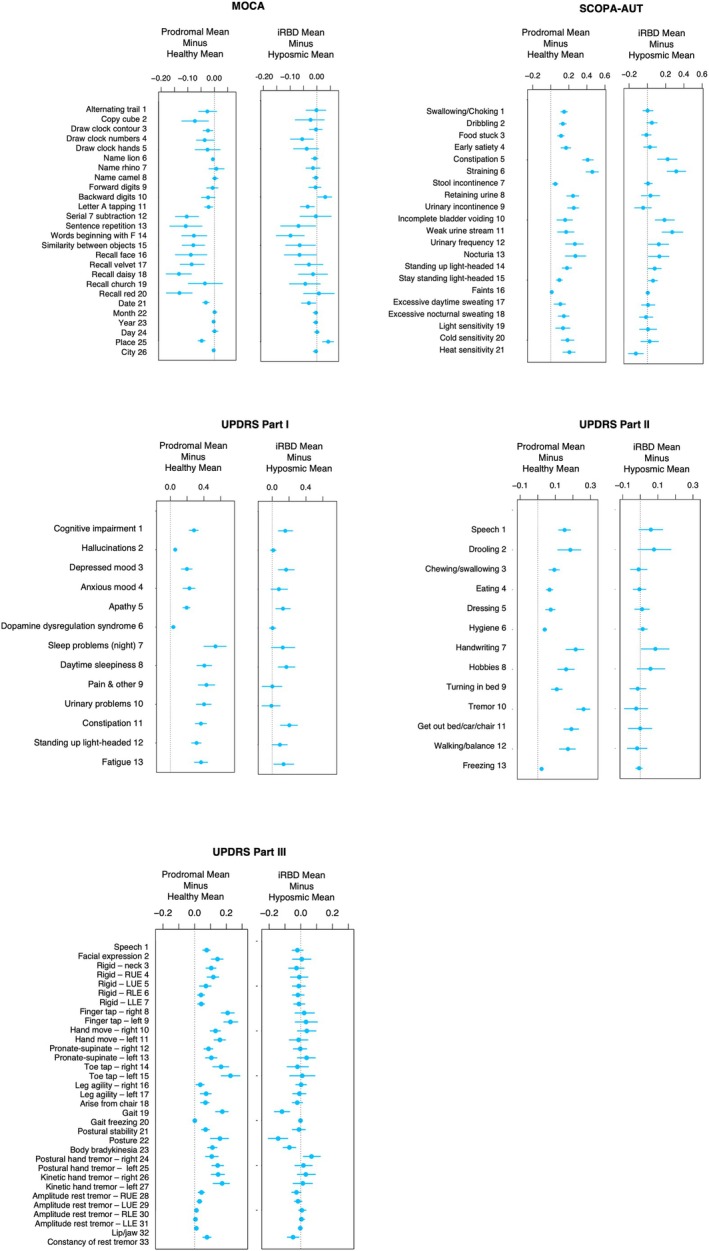
Item‐level mean differences in questionnaire responses between groups. Questions are shown on the y‐axis and mean difference (first group minus comparator) with 95% confidence intervals on the x‐axis.

Figure 3 presents item‐level mean differences (with 95% confidence intervals) in question responses between groups, illustrating which items contributed most to the overall between‐group differences in total scores. Adjustments for age and sex do not alter the ranking of these contributions. For the comparison between healthy controls and the combined prodromal cohort, the largest item‐level differences were: MoCA—delayed recall; sentence repetition, SCOPA‐AUT—straining to pass stools; constipation; nocturia, MDS‐UPDRS Part I—night‐time sleep problems; pain and other sensations; urinary problems, Part II—tremor; handwriting; rising from a low seated or lying position, and Part III—toe tapping [left]; finger tapping [left]; finger tapping [right]. For comparisons showing significant differences between iRBD and hyposmic participants (higher in iRBD), the largest item‐level differences were in SCOPA‐AUT—straining to pass stools; weak urine stream; constipation and MDS‐UPDRS Part I—constipation; depression; daytime sleepiness.

## 4 Discussion

Our results demonstrate that significant early differences are present between two enriched prodromal LBD cohorts, PSG‐confirmed iRBD and iRBD‐negative hyposmia with abnormal DAT‐SPECT, before the emergence of a diagnostic motor syndrome. Both cohorts showed significantly worse cognitive, autonomic, and motor performance compared with healthy controls, consistent with early neurodegenerative pathology. Critically, the two prodromal groups themselves diverged in autonomic symptom burden but not in cognitive or motor measures, a pattern with implications for understanding prodromal heterogeneity and the design of future neuroprotective trials.

Research into prodromal LBD subtypes remains limited, in part due to the challenging nature of identifying and recruiting participants prior to diagnosis. This is particularly true for hyposmia, which has lower specificity for prodromal LBD than iRBD when considered in isolation [5, 25]. This study uniquely captured a DAT‐SPECT enriched cohort of iRBD‐negative hyposmia participants, thereby enriching for participants more likely to have prodromal LBD than hyposmia alone.

iRBD participants scored significantly higher than those with hyposmia in both SCOPA‐AUT and MDS‐UPDRS Part I scores. Given the substantial overlap in question content of these two clinical tools, the concurrence of findings is expected. The excess of autonomic symptoms in iRBD relative to the hyposmic cohort aligns with the brain‐first/body‐first framework, in which iRBD is considered a marker of the body‐first subtype characterized by early peripheral autonomic involvement [6, 11, 26, 27]. That this divergence is already apparent at the prodromal stage suggests that these two phenotypes may reflect distinct pathological routes to LBD rather than different points on a single trajectory. Our item‐level analysis further identifies constipation and urinary symptoms as the specific autonomic domains driving this difference, consistent with early enteric and lower urinary tract denervation preceding central neurodegeneration. Given the modest effect sizes, these differences are best interpreted as group‐level trends that may inform stratification in future clinical trials rather than individually discriminating features.

No between‐group differences were observed for MoCA or for MDS‐UPDRS Parts II and III. As these clinical assessments primarily capture cognitive performance and motor impairment, the absence of differences may reflect limited separation at this early stage, or genuinely similar trajectories between cohorts. Longitudinal follow‐up to phenoconversion will be needed to determine whether divergence emerges over time and to characterize subsequent clinical progression.

In MDS‐UPDRS Part I, daytime sleepiness was identified as a significant question. Given iRBD is a sleep disorder, it is unsurprising that the iRBD group showed higher scores of sleep‐related symptoms. It should be acknowledged that this may reflect an unaccounted confounding variable in this analysis, potentially contributing to the observed group differences in MDS‐UPDRS Part I.

A limitation of this study's methodology is the assumption that the iRBD and hyposmic groups are at a comparable stage in the disease process. Differences in time to phenoconversion between groups could, in principle, account for observed differences in symptom burden. We mitigated this by analyzing the earliest available assessments for each participant, and the cohorts were of similar age, providing an approximately age‐matched snapshot. DAT‐SPECT striatal binding ratios (SBRs) were broadly comparable between groups, suggesting a similar degree of underlying dopaminergic deficit at the time of assessment. Notably, the hyposmia cohort showed slightly lower DAT binding than the iRBD cohort, consistent with greater nigrostriatal involvement, yet symptom burden was higher in iRBD. This pattern argues against simple differences in disease stage as the sole explanation for the between‐group findings. Longitudinal follow‐up to phenoconversion within PPMI will be important to test this directly.

It is worth highlighting that hyposmia was defined solely by UPSIT performance, without systematic exclusion of peripheral causes of olfactory dysfunction (such as viral infection or nasal trauma). However, the requirement and presence of concurrent dopaminergic deficit on DAT‐SPECT in this cohort strongly suggest that hyposmia is likely neurodegenerative rather than peripheral in origin. Additionally, polysomnography was not performed in the hyposmia cohort, meaning some participants may have had undetected subclinical RBD. However, this would attenuate rather than inflate between‐group differences, making our findings conservative.

By design, we defined mutually exclusive cohorts, iRBD‐positive without hyposmia and iRBD‐negative with hyposmia, to isolate two clinically distinct prodromal phenotypes and minimize phenotypic overlap between groups. This approach may limit the generalizability of our findings to the full prodromal LBD spectrum, as participants with both features were not represented in our analytic sample. However, given that olfactory dysfunction in iRBD predicts phenoconversion to overt synucleinopathy, participants with both iRBD and hyposmia likely represent a more advanced stage of prodromal LBD [28, 29]. Including these participants in the iRBD cohort would therefore bias the sample toward more advanced prodromal disease, rather than the early‐stage phenotype our study aimed to characterize. Nevertheless, we repeated the analysis including iRBD participants with concurrent hyposmia, which did not materially alter any findings (Tables S1 and S2). However, only 12 such participants had complete data, limiting the power of this comparison.

Although iRBD and hyposmia with DAT deficit both confer a high probability of underlying synucleinopathy, neither marker guarantees phenoconversion and a proportion of participants in both cohorts may never develop a clinically diagnosed Lewy body disorder. However, both cohorts differed significantly from healthy controls across multiple domains, and the differences between the hyposmic and iRBD cohorts are domain‐specific rather than uniform, consistent with distinct prodromal phenotypes rather than dilution by non‐synucleinopathy cases. Longitudinal PPMI data will permit future analysis of phenoconversion rates and endpoint diagnoses across these prodromal groups.

A minority of iRBD participants may ultimately phenoconvert to multi‐system atrophy rather than PD or DLB, which could contribute to the higher autonomic symptom burden observed in the iRBD cohort. However, MSA accounted for only 4.5% of phenoconversions in the largest multicentre iRBD cohort to date, making substantial distortion of the observed autonomic signal unlikely [13].

The clinical relevance of these findings extends to the design of neuroprotective trials targeting prodromal populations. The observation that both prodromal cohorts scored significantly worse than healthy controls across all clinical domains confirms that individuals with iRBD or hyposmia with DAT deficit represent clinically identifiable at‐risk populations amenable to early intervention. That these differences are detectable using standard clinical instruments at the prodromal stage supports the feasibility of recruiting and monitoring enriched prodromal cohorts in trial settings. Furthermore, the finding that autonomic measures differentiate between the two prodromal subtypes, while cognitive and motor assessments do not, has practical implications for trial stratification: MoCA and MDS‐UPDRS Parts II and III may not be informative for distinguishing prodromal subtypes, whereas autonomic symptom profiles, MDS‐UPDRS Part I and SCOPA‐AUT, may have value for subtyping within prodromal trial populations.

In conclusion, we found reproducible clinical differences between two enriched prodromal cohorts. iRBD‐positive participants had a higher autonomic symptom burden than the hyposmia with DAT‐deficit cohort, while cognitive and motor measures were broadly similar at this early stage.

## Author Contributions

**Jacopo Pasquini:** conceptualization, methodology, formal analysis, writing – original draft, writing – review and editing, project administration. **Luke Vikram Banerjee:** conceptualization, methodology, software, data curation, formal analysis, visualization, project administration, writing – original draft, writing – review and editing, investigation. **Robin Henderson:** software, data curation, methodology, visualization, formal analysis. **Kirstie N. Anderson:** conceptualization, methodology, writing – review and editing, supervision, writing – original draft. **Nicola Pavese:** conceptualization, methodology, writing – review and editing, supervision, writing – original draft.

## Funding

The authors have nothing to report.

## Disclosure

PPMI—a public‐private partnership—is funded by the Michael J. Fox Foundation for Parkinson's Research and funding partners, including 4D Pharma, Abbvie, AcureX, Allergan, Amathus Therapeutics, Aligning Science Across Parkinson's, AskBio, Avid Radiopharmaceuticals, BIAL, BioArctic, Biogen, Biohaven, BioLegend, BlueRock Therapeutics, Bristol‐Myers Squibb, Calico Labs, Capsida Biotherapeutics, Celgene, Cerevel Therapeutics, Coave Therapeutics, DaCapo Brainscience, Denali, Edmond J. Safra Foundation, Eli Lilly, Gain Therapeutics, GE HealthCare, Genentech, GSK, Golub Capital, Handl Therapeutics, Insitro, Jazz Pharmaceuticals, Johnson & Johnson Innovative Medicine, Lundbeck, Merck, Meso Scale Discovery, Mission Therapeutics, Neurocrine Biosciences, Neuron23, Neuropore, Pfizer, Piramal, Prevail Therapeutics, Roche, Sanofi, Servier, Sun Pharma Advanced Research Company, Takeda, Teva, UCB, Vanqua Bio, Verily, Voyager Therapeutics, the Weston Family Foundation, and Yumanity Therapeutics.

## Ethics Statement

This study used de‐identified data from the Parkinson's Progression Markers Initiative (PPMI) database. PPMI was approved by the institutional review board/ethics committee at each participating site, and all participants provided written informed consent. Data were accessed following approval of our PPMI data use application and in accordance with the PPMI Data Use Agreement.

## Conflicts of Interest

N.P. and K.N.A. receive grant funding from the Michael J Fox Foundation to run the Parkinson's Progression Markers Initiative study in Newcastle upon Tyne. N.P. has served on advisory boards for BIAL, F. Hoffmann–La Roche Ltd. and AbbVie and has received research funding from the Independent Research Fund Denmark, the Danish Parkinson's Disease Association, Parkinson's UK, GE HealthCare, the Multiple System Atrophy Trust, F. Hoffmann‐La Roche, and the Michael J. Fox Foundation for Parkinson's Research. K.N.A. has served on advisory boards for Janssen‐Cilag and Idorsia, has received grant funding from the Closing the Gap mental health network, and has received speaker fees from Bioprojet, Flynn Pharma and Idorsia. The other authors declare no conflicts of interest.

## Supporting information


**Table S1:** Characteristics of the iRBD Cohort inclusive of iRBD participants with concurrent Hyposmia.
**Table S2:** Age‐ and Sex‐Adjusted Group Comparisons inclusive of iRBD participants with concurrent Hyposmia.

## Data Availability

The data analyzed in this study are available from the Parkinson's Progression Markers Initiative (PPMI) repository to qualified researchers following application approval and acceptance of the PPMI Data Use Agreement. The data are not publicly available without restriction due to these access controls.
