# Supplementary material for: Decoding Prodromal Lewy Body Disease: Clinical Differences Between Isolated REM Sleep Behavior Disorder and Hyposmia With Dopamine Transporter Deficit
Source: Eur J Neurol. 2026 Aug 6;33(8):e70724. doi: 10.1111/ene.70724 (PMC13446031; doi:10.1111/ene.70724)
Supplement: Supplementary file 1 [file ENE-33-e70724-s001.docx]

| **Characteristic** | **iRBD**  **(n = 372)** |
| --- | --- |
| Age at Visit  (years) | 67.7 (6.1) |
| Sex | 82 females  290 males |
| MoCA | 26.3 (2.8) |
| SCOPA-AUT | 10.6 (6.9) |
| MDS-UPDRS Part I | 8.1 (5.8) |
| MDS-UPDRS Part II | 2.5 (3.6) |
| MDS-UPDRS Part III | 4.8 (5.4) |

**Supplementary Table 1. Characteristics of the iRBD Cohort inclusive of iRBD participants with concurrent Hyposmia**

*Data values appear as unadjusted means (standard deviation). This iRBD group includes 12 additional participants with concurrent hyposmia.*

| **Outcome** | **Comparison** | **Mean**  **Difference** | **p-value** | **Cohen’s d** |
| --- | --- | --- | --- | --- |
| **MoCA** | Healthy vs Prodromal | −1.08 | <0.001 | −0.47 |
|  | iRBD vs Hyposmia | −0.19 | 0.192 | −0.08 |
| **SCOPA-AUT** | Healthy vs Prodromal | 3.43 | <0.001 | 0.56 |
|  | iRBD vs Hyposmia | 1.83 | <0.001 | 0.30 |
| **MDS-UPDRS**  **Part I** | Healthy vs Prodromal | 3.57 | <0.001 | 0.70 |
|  | iRBD vs Hyposmia | 1.67 | <0.001 | 0.33 |
| **MDS-UPDRS**  **Part II** | Healthy vs Prodromal | 1.53 | <0.001 | 0.47 |
|  | iRBD vs Hyposmia | 0.36 | 0.084 | 0.11 |
| **MDS-UPDRS**  **Part III** | Healthy vs Prodromal | 2.91 | <0.001 | 0.52 |
|  | iRBD vs Hyposmia | −0.42 | 0.233 | −0.07 |

**Supplementary Table 2. Age- and Sex-Adjusted Group Comparisons inclusive of iRBD participants with concurrent Hyposmia**

*Statistical methods are identical to the primary analysis.*
